# Risk factors for the effect of anticoagulant and antiplatelet agents on perioperative blood loss following proximal femoral fractures

**DOI:** 10.1097/MD.0000000000004120

**Published:** 2016-07-08

**Authors:** Yusuke Akaoka, Hiroshi Yamazaki, Hiroyuki Kodaira, Hiroyuki Kato

**Affiliations:** a Department of Orthopedic Surgery, Shinshu University School of Medicine,Matsumoto, Nagano,Japan; b Department of Orthopedic Surgery, Aizawa Hospital, Matsumoto, Nagano, Japan.

**Keywords:** anticoagulants, antiplatelet agents, hemoglobin level, operative time, perioperative blood loss, platelet count, proximal femoral fracture

## Abstract

To examine the effect of oral anticoagulant and antiplatelet agents on perioperative blood loss following proximal femoral fractures and to identify the risk factors associated with perioperative blood loss.

Retrospective cross-sectional study.

In a retrospective cross-sectional study, we treated 334 consecutive patients with proximal femoral fractures (100 who received anticoagulant or antiplatelet drugs and 234 who did not) and an overall mean age of 85.5 years (standard deviation 8.2 years). We performed retrospective multivariate analysis to determine the independent factors related to perioperative decreases in the hemoglobin (Hb) level, a proxy for blood loss.

Multivariate analysis confirmed that anticoagulant or antiplatelet drugs significantly affected decreases in the Hb level (regression coefficient [RC], 0.61; 95% confidence interval [CI], 0.14–1.08; *P* = 0.01). In addition to anticoagulant or antiplatelet drugs, multivariate analysis confirmed that the fracture type (Orthopedic Trauma Association classification A2: RC, 1.19; 95% CI, 0.71–1.67; *P* < 0.01; A3: RC, 2.47; 95% CI, 1.41–3.53; *P* < 0.01), platelet count (RC, −0.08; 95% CI, −0.12 to −0.04; *P* < 0.01), and operative time (RC, 0.02; 95% CI, 0.004–0.03; *P* = 0.01) affected the decreases in Hb level.

The use of anticoagulants and antiplatelet agents is an independent risk factor for perioperative blood loss following proximal femoral fractures. Fracture type, platelet count, and operative time also affect perioperative blood loss. The fracture type was the greatest contributing factor to perioperative blood loss.

Level of evidence grade: Prognostic level III.

## 1 Introduction

Currently, there is no consensus about the effects of anticoagulant and antiplatelet agents on perioperative blood loss following proximal femoral fractures in elderly patients. According to the guidelines published by the American Academy of Orthopedic Surgeons in 2014,^[1–6]^ evidence regarding perioperative bleeding with aspirin and/or clopidogrel intake is limited. There have been contradictory reports of increased blood loss with aspirin^[4,7]^ and clopidogrel^[2]^ and reports that suggest no effects of these drugs on blood loss.^[5,8]^ Most previous studies have only included bivariate analyses that compared blood loss and transfusion requirements with nonexposure or exposure to anticoagulant/antiplatelet drugs; other factors were not examined in these studies.^[2,4,5,7,8]^ The discrepancy in previous findings may be because perioperative bleeding can be affected by factors beyond the administration of oral anticoagulant and antiplatelet drugs, such as the fracture type, platelet count, and surgery time. Only 2 reports describing an association between blood loss and unexposed or exposed anticoagulant/antiplatelet using multivariable analysis have been published.^[6,9]^ The aim of this study was twofold: to determine whether anticoagulant and antiplatelet agents (aspirin, clopidogrel, etc.) affect perioperative blood loss following proximal femoral fractures, and to identify the risk factors for blood loss.

## 2 Patients and methods

### 2.1 Patients

We conducted a retrospective cross-sectional study of 362 consecutive patients who underwent surgical treatment for trochanteric fractures at our institute from March 2011 to May 2013. Patients with artificial dialysis (n = 9), moderate or severe postoperative pneumonia (n = 13), recently confirmed bleeding ulcer (n = 5), and a refracture from a fall during hospitalization (n = 1) were excluded from the study population due to their potential influence on postoperative hemoglobin (Hb) levels. After these exclusions were applied, the final study population consisted of 334 patients.

Informed consent was obtained from all patients. The study was approved by the Aizawa Hospital Institutional Review Board.

### 2.2 Study size

The calculation of sample size was set to 5% level of significance and 80% study power. When the clinical significance of reduction in Hb level (10% of the mean decrease in Hb level) and standard deviation were set at 0.39 and 1.00, respectively, the necessary number of data were 156 for the control group and 78 for the test group.

### 2.3 Preoperative management, anesthesia, and surgical procedure

The administered group continued oral administration of anticoagulant and/or antiplatelet drugs before surgery and did not undergo heparinization or neutralization by vitamin K administration. There was no difference in the treatment method for providing preoperative care and rehabilitation protocols between the 2 groups. Because we believe the advantages of early operation supersede those of preoperative blood loss, an early operation was performed regardless of whether the patients were taking oral anticoagulants and antiplatelets. All patients in the administered group underwent general anesthesia. In consultation with an anesthesiologist, 146 of the 234 patients in the nonadministered group underwent spinal anesthesia. Eighty-eight patients with decreased cognitive ability in the nonadministered group underwent general anesthesia, taking into account their inability to remain calm intraoperatively. Surgical treatments were performed by surgeons with varying years of experience. They were divided into 3 groups depending on the surgeon's experience (1–3 years, 4–6 years, over 7 years) in surgical treatment for trochanteric fractures. Trochanteric fractures were treated using nail devices or dynamic hip screws. A summary of implants is provided in Table 1.

**Table 1:**
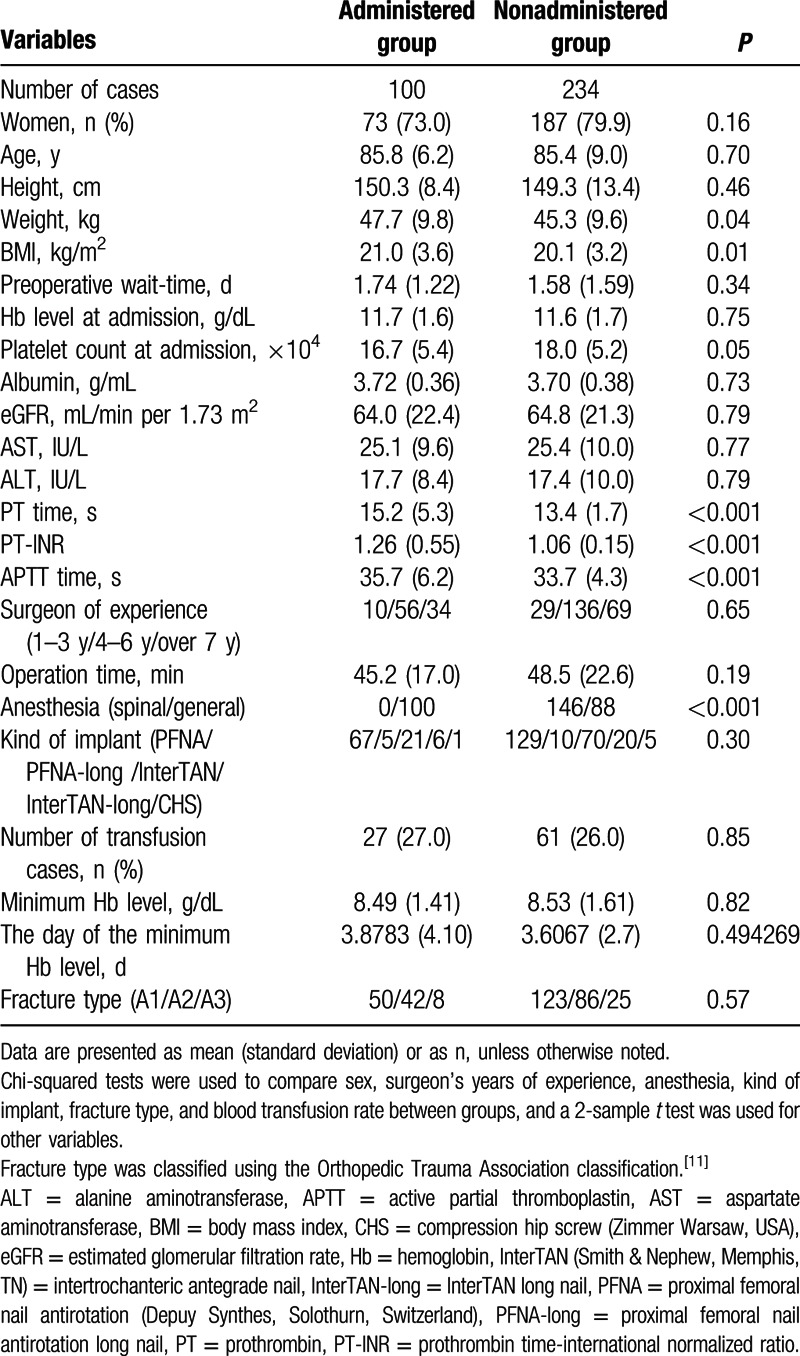
Patient demographics.

### 2.4 Outcomes

The main outcome of this study was the decrease in the Hb level, which was defined as the difference between the Hb level at admission and the minimum Hb level. Hb levels were usually assessed at admission, preoperatively, and on postoperative days 1, 2, 4, 7, and 14. The conversion equation for decrease in Hb level for blood transfusion cases is as follows: 
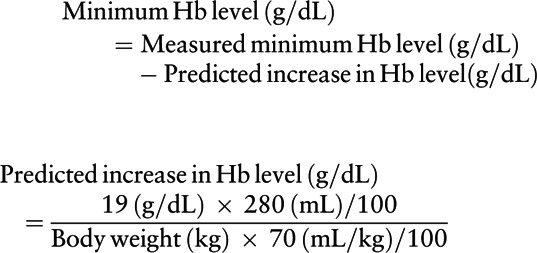


The circulating blood volume is 70 mL/kg, and the Hb level of the packed red blood cells is 19 g/dL and the 2 U are 280 mL.^[10]^

### 2.5 Statistical analyses

We determined the factors related to the decrease in Hb level (response variable) using multivariate analysis by setting the entry method, 1 of which is forced entry. The following explanatory variables were selected based on their potential to affect clinical perioperative bleeding: whether or not drugs were administered, age, sex, weight, platelet count, prothrombin time (PT), active partial thromboplastin time (APTT), preoperative wait time, fracture type (Orthopedic Trauma Association [OTA] classification),^[11]^ operative time, surgeon experience, and type of implant. PT time and APTT were separated into 2 groups by PT < 15 s and APTT < 40 s. Surgeon's years of experience were classified into 3 groups (experience of 1–3 years, 4–6 years, and over 7 years in surgical treatment trochanteric fractures). Fracture type was classified using OTA classification,^[11]^ and the fractures were classified into 3 groups (A1, A2, and A3). The implants were classified into 4 groups: intertrochanteric antegrade nail (InterTAN: Smith & Nephew, Memphis, TN), compression hip screw (CHS: Zimmer Warsaw, USA), proximal femoral nail antirotation long nail (PFNA-long: DePuy Synthes, West Chester, PA), and intertrochanteric antegrade nail long nail (InterTAN-long). Analysis was performed based on 1 to 3 years, A1 type fractures, and proximal femoral nail antirotation (PFNA) implants.

Multivariate analysis was repeated for the drug-administered group in order to determine the effect of the drugs by subgroup.

We defined statistical significance as *P* < 0.05. All analyses were performed with commercially available software (Stat Flex version 5.0, Artech, Inc., Osaka, Japan).

## 3 Results

### 3.1 Patient demographics

Demographic data are shown in Table 1.

Between the administered and nonadministered groups, there were no statistical differences for sex, age, height, preoperative wait time, Hb level at time of admission, platelet count, albumin level, estimated glomerular filtration rate, aspartate aminotransferase level, alanine aminotransferase level, surgeon experience, operative time, type of implant, minimum Hb level, or fracture type. Transfusion was required in 27 cases in the administered group and 61 in the nonadministered group (nonsignificant). Weight, body mass index, PT time, PT-international normalized ratio, APTT, and anesthesia were significantly different. The mean minimum Hb level was recorded postoperatively at 3.72 (±3.1) days (Table 1). Out of 334 patients, 100 were classified into the administered group. The administered group either received a single or dual drug therapy. Single drug therapy cases included 37 patients receiving Aspirin as an antiplatelet agent, 27 receiving antiplatelet agents other than Aspirin (consisting of clopidogrel, 10; cilostazol, 10; ticlopidine, 7), and 18 receiving warfarin as an anticoagulant. There were 18 dual drug therapy cases of which 4 patients received a combination of aspirin and warfarin, and 14 patients received a combination of an antiplatelet with other agents (Table 2).

**Table 2:**
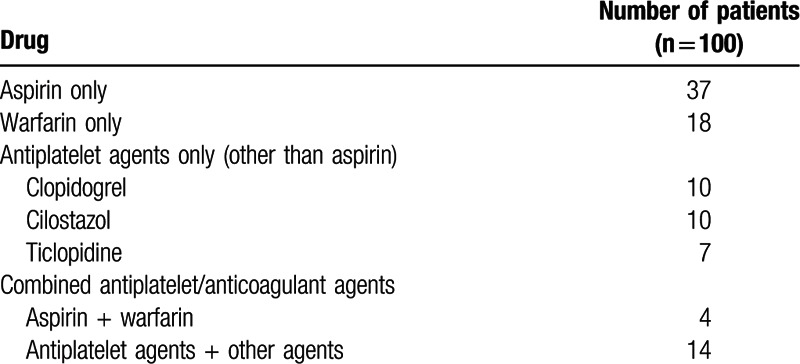
Anticoagulant and antiplatelet agents taken by patients in the administered group.

### 3.2 Outcomes

For all patients, the mean decrease in Hb level was 3.90 (±2.2) g/dL. The univariate analysis (crude) showed that drug administration nonsignificantly affected the decrease in Hb levels (regression coefficient [RC], 0.36; 95% confidence interval [CI], −0.15 to 0.08; *P* = 0.88). The univariate analysis showed that platelet count (RC, −0.08; 95% CI, −0.73 to −0.82; *P* < 0.01), APTT (RC, −0.04; 95% CI, −0.009 to −0.005; *P* < 0.01), fracture type (A2: RC, 0.82; 95% CI, 0.35–1.30; *P* < 0.01, A3: RC, 2.50; 95% CI, 1.75–3.24; *P* < 0.01), operative time (RC, 0.03; 95% CI, 0.02–0.04; *P* < 0.01), and type of implant (InterTAN: RC, 0.57; 95% CI, 0.04–1.10; *P* = 0.04, PFNA-long: RC, 1.47; 95% CI, 0.35–2.60; *P* = 0.01, InterTAN-long: RC, 2.24; 95% CI, 1.39–3.09; *P* < 0.01) affected the decrease in Hb levels. Age, sex, weight, PT, preoperative wait time, surgeon experience, and type of implant (CHS) did not affect the decrease in Hb levels. Multiple regression analysis (adjusted) showed that drug administration significantly affected the decrease in Hb levels (RC, 0.61; 95% CI, 0.14–1.08; *P* = 0.01). The multiple regression analysis also showed that platelet count (RC, −0.08; 95% CI, −0.04 to −0.12; *P* < 0.01), fracture type (A2: RC, 1.19; 95% CI, 0.70–1.68; *P* < 0.01, A3: RC, 2.48; 95% CI, 1.42–3.54; *P* < 0.01), and operative time (RC, 0.02; 95% CI, 0.008–0.030; *P* = 0.01) affected the decrease in Hb levels. Age, sex, weight, PT, APTT, preoperative wait time, surgeon experience, and type of implant did not affect the decrease in Hb levels (Table 3).

**Table 3:**
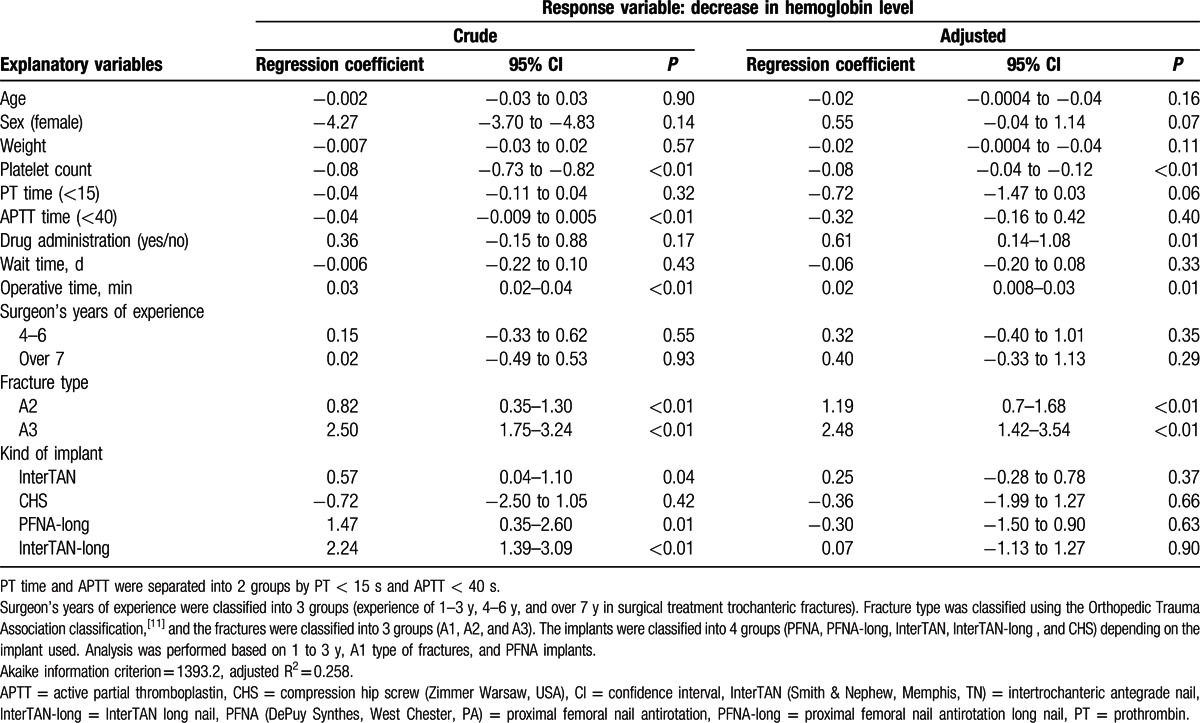
Results of univariate and multiple regression analyses.

In a multiple regression analysis of subgroups from the drug-administered group, there was no significant difference in Hb decrease according to the types of administered drugs (Table 4).

**Table 4:**
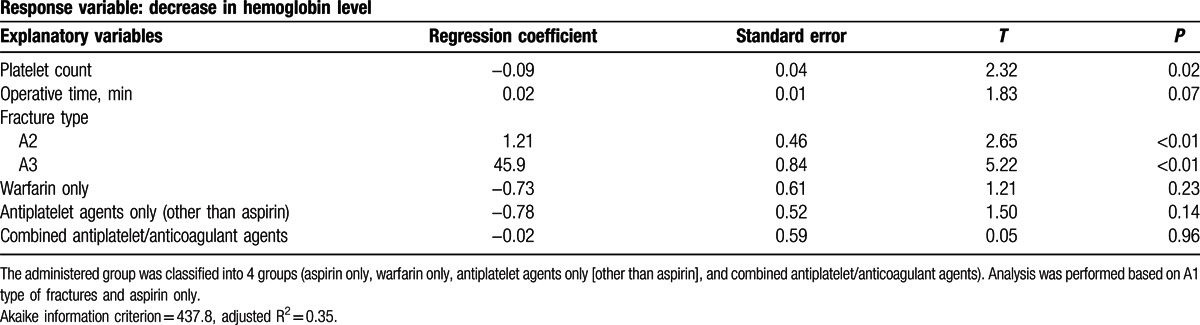
Results of a subgroup multiple regression analysis.

## 4 Discussion

In the present study, we did not require a waiting period after drug discontinuation for the anticoagulant and antiplatelet administered group and so the surgery was performed as early as possible for both groups without heparinization or neutralization using vitamin K. The results from the univariate regression analysis showed that the administration of anticoagulant and antiplatelet drugs nonsignificantly affected the decrease in Hb level, with the administered group showing a 0.36 g/dL greater decrease in Hb level (95% CI, −0.15 to 0.08). The decrease in Hb level was significantly affected by fracture type, platelet count, APTT, operative time, and type of implant. However, confounder-adjusted results from the multiple regression analysis showed that the administration of anticoagulant and antiplatelet drugs significantly affected the decrease in Hb level, with the administered group showing a 0.61 g/dL greater decrease in Hb level (95% CI, 0.14–1.08).

Other than anticoagulant and antiplatelet drug administration, the decrease in Hb level was also significantly affected by fracture type, platelet count, and operative time. In particular, fracture type was the most significant factor, which showed a 1.19 g/dL (95% CI, 0.71–1.67) greater decrease in Hb level in OTA classification type A2 fractures and a 2.47 g/dL (95% CI, 1.41–3.53) greater increase in type A3 fractures compared with type A1 fractures. We found that the PT and APTT measured to examine the coagulation system had no influence on perioperative bleeding. There was no significant difference in the Hb level decrease based on an analysis of the anticoagulant and antiplatelet agent subtypes.

Previous studies have described an association between blood loss and nonexposure or exposure to anticoagulant/antiplatelet agents. Manning et al^[4]^ surgically treated 89 people with femoral neck fractures using hemi-arthroplasty or dynamic hip screws. They reported that the aspirin-administered group had a lower preoperative Hb level than the nonadministered group. Similarly, although there was no significant difference in the postoperative decrease in the Hb level, aspirin-administered patients were significantly more likely to have postoperative anemia and greater blood transfusion rates compared to the nonadministered patients.

Chechik et al^[2]^ surgically treated a total of 88 patients using hemi-arthroplasty for displaced subcapital (Garden 3 and 4) hip fractures and a dynamic hip screw or proximal femoral nail for proximal trochanteric fractures, and reported that patients in the continuous clopidogrel or combined clopidogrel and aspirin groups had significantly greater blood loss than those in the aspirin-administered and nonadministered groups. They also reported that blood loss was influenced by operative time.

Collinge et al^[8]^ used a side plate and screw or nail device for arthroplasty for femoral neck fractures and proximal trochanteric fractures in 1036 patients. They reported that there was no significant difference in blood loss between the nonadministered and administered groups taking clopidogrel, aspirin, or warfarin.

A major shortcoming in these reports is their failure to examine for confounding factors due to their simple 2-group (multigroup) comparison of blood loss and transfusion requirements with nonexposure or exposure to anticoagulant/antiplatelet agents. We believe that perioperative blood loss is affected not only by the administration of anticoagulant and antiplatelet agents but by a multitude of factors, including fracture type, platelet count, and operative time. Perioperative bleeding could be significantly affected by fracture site, fracture type, and surgical methods, though there is a paucity of research on these relationships. The lack of consensus for the effect of oral anticoagulant and antiplatelet therapy might be due to the differences in patient-related factors and the lack of confounding factor analysis.

To our knowledge, there are only 2 reports describing an association between transfusion rate and nonexposure/exposure to anticoagulant/antiplatelet agents using multivariable analysis.

Hossain et al^[6]^ surgically treated 92 patients with femoral neck fractures using hemi-arthroplasty. They reported that there was no significant difference in required postoperative transfusion between the nonadministered and administered groups taking clopidogrel (*P* = 0.37). The covariate adjusted risk ratio for transfusion while receiving clopidogrel treatment was 3.96 (95% CI, 0.40–39.68).

Madsen et al^[9]^ reported a factor associated with the transfusion of 986 consecutive hip fracture patients in multivariable analysis. Factors associated with the likelihood of requiring transfusion were increasing age, the American Society of Anesthesiologists’ physical status classification ≥3, being admitted from own home, extracapsular fractures, decreasing admission Hb levels, and use of platelet inhibitors were all significantly associated with the risk of receiving a red blood cell transfusion.

The decision to perform a transfusion is made comprehensively, based on the patient's vital signs and medical history; therefore, it is not regarded as an objective assessment item despite its use in clinical assessment. In the present study, we used Hb levels, which was an objective method to measure the outcome and presence of confounding factors on perioperative bleeding by conducting a multivariate regression analysis. The results showed that administration of anticoagulant and antiplatelet drugs was associated with a greater decrease in Hb levels, suggesting that when various confounding factors are excluded, anticoagulant and antiplatelet drug administration increases perioperative blood loss.

There is no consensus for the cessation period for anticoagulant and antiplatelet agents prior to the treatment of proximal femoral fractures. Depending on the drug type and taking into account drug pharmacokinetics, there is a need for a drug cessation period of 3 to 14 days; for administered patients, the drug cessation period results in an extension of their preoperative wait time. However, early surgery may minimize complications and shorten the hospital stay.^[12–14]^ According to the Management of Hip Fractures in the Elderly Evidence-Based Clinical Practice Guidelines,^[1]^ moderate evidence supports that hip fracture surgery within 48 h of admission is associated with better outcomes. Moreover, there is also a concern that suspending anticoagulant and antiplatelet drug administration may cause its own set of complications. Maulaz et al^[15]^ reported that the odds ratio for strokes after suspending aspirin intake is 3.4, and Servin^[16]^ reported that aspirin administration cessation for patients with coronary heart disease is 2 to 4 times more likely to result in mortality or myocardial infarction. The complications from an extended preoperative wait time due to drug cessation in patients taking anticoagulant and antiplatelet agents, as well as the possibility of cerebral and myocardial infarction caused by drug cessation, should be considered. Although there was no clinical equivalence based on the equivalence and noninferiority tests, we believe that this is permissible considering the patient's alleviation of burdens, improvement in functional prognosis, and early discharge.

## 5 Limitations

The present study had a number of limitations. First, the decrease in Hb level (defined as the difference between the Hb value at admission and the lowest Hb value) was used to assess perioperative bleeding. Measuring Hb level is easy, but results may be affected by patient-specific variables such as dehydration or physique. The patient's condition cannot be ascertained by the Hb value alone, and although it can help determine whether a transfusion is needed, it is not definitive, and may be somewhat unsuitable from the viewpoint of clinical assessment. As described in previous studies,^[6,9]^ there is also a method of assessing the effect of anticoagulant and antiplatelet drug administration from the presence or absence of transfusion. As described already, the decision to perform transfusion is made comprehensively and is not regarded as an objective assessment item. It is also difficult to determine the entire perioperative bleeding amount by measuring the intraoperative bleeding and amount of postoperative blood lost to drainage. In light of the above points, the Hb value was used as a parameter for objectively assessing the extent to which perioperative bleeding is affected by anticoagulant antiplatelet drugs.

The method of anesthesia was significantly different between the 2 groups. It seems possible that the difference in blood loss during surgery was affected by the patient's blood pressure control, depending on the method of anesthesia. However, this issue is not specific to our facility; in Japan, the problems posed by the number of anesthetists and anesthesiologists make it difficult to operate all cases under general anesthesia or anesthetic management. Thus, spinal anesthesia was widely prevalent in the nonadministered group in our study.

Because our objective was to determine the validity of our results, we used a regression equation to examine the response variable in multiple regression and validation analyses. With the adjusted R^2^ at 0.35 and an Akaike information criterion of 1393.2, the regression equation is not adequate to predict the decrease in Hb level. Therefore, we believe that the prediction of intraoperative bleeding should be determined comprehensively according to the surgeon's own experience.

Future research should compare the complications that can be caused by anticoagulant and antiplatelet drug continuation with those caused by their discontinuation in patients with proximal femoral fractures with more detailed investigation of outcomes, including mortality.

## 6 Conclusions

The use of anticoagulant and antiplatelet agents was an independent risk factor for perioperative blood loss following proximal femoral fractures. Additionally, the fracture type, sex, platelet count, and operative time affected perioperative blood loss. Particularly, the fracture type greatly influenced bleeding.
